# On the Performance of Linear Decreasing Inertia Weight Particle Swarm Optimization for Global Optimization

**DOI:** 10.1155/2013/860289

**Published:** 2013-10-31

**Authors:** Martins Akugbe Arasomwan, Aderemi Oluyinka Adewumi

**Affiliations:** School of Mathematics, Statistics, and Computer Science, University of Kwazulu-Natal, Private Bag X54001, Durban 4000, South Africa

## Abstract

Linear decreasing inertia weight (LDIW) strategy was introduced to improve on the performance of the original particle swarm optimization (PSO). However, linear decreasing inertia weight PSO (LDIW-PSO) algorithm is known to have the shortcoming of premature convergence in solving complex (multipeak) optimization problems due to lack of enough momentum for particles to do exploitation as the algorithm approaches its terminal point. Researchers have tried to address this shortcoming by modifying LDIW-PSO or proposing new PSO variants. Some of these variants have been claimed to outperform LDIW-PSO. The major goal of this paper is to experimentally establish the fact that LDIW-PSO is very much efficient if its parameters are properly set. First, an experiment was conducted to acquire a percentage value of the search space limits to compute the particle velocity limits in LDIW-PSO based on commonly used benchmark global optimization problems. Second, using the experimentally obtained values, five well-known benchmark optimization problems were used to show the outstanding performance of LDIW-PSO over some of its competitors which have in the past claimed superiority over it. Two other recent PSO variants with different inertia weight strategies were also compared with LDIW-PSO with the latter outperforming both in the simulation experiments conducted.

## 1. Introduction

The idea of Particle Swarm Optimization (PSO) stems from biology where a swarm of birds coordinates itself in order to achieve a goal. When a swarm of birds looks for food, its individuals will spread in the environment and move around independently with some degree of randomness which enables it to discover food accumulations. After a while, one of them will find something digestible and, being social, communicates this to its neighbors. These can then approach the source of food, thus leading to the convergence of the swarm to the source of food. Following this analogy, PSO was largely derived from sociopsychology concept and transferred to optimization [1], where each particle (bird) uses the local information regarding the displacement of its reachable closer neighbors to decide on its own displacement, resulting to complex and adaptive collective behaviors.

Since the inception of PSO technique, a lot of work has been done by researchers to enhance its efficiency in handling optimization problems. One such work is the introduction of linear decreasing inertia weight (LDIW) strategy into the original PSO to control its exploration and exploitation for better performance [2–4]. However, LDIW-PSO algorithm from the literature is known to have the shortcoming of premature convergence in solving complex (multipeak) problems due to lack of enough momentum for particles to do exploitation as the algorithm approaches its terminal point. The challenge of addressing this shortcoming has been on for a long time and has attracted much attention of researchers in the field of global optimization. Consequently upon this, many other inertia weight PSO variants have been proposed [2, 5–16], with different levels of successes. Some of these variants have claimed better performances over LDIW-PSO, thereby making it look weak or inferior. Also, since improving on the performance of PSO is an area which still attracts more researchers, this paper strives to experimentally establish the fact that LDIW-PSO is very much efficient if its parameters, like velocity limits for the particles, are properly set. Using the experimentally obtained values for particle velocity limits in LDIW-PSO, results show that LDIW-PSO outperformed other PSO variants adopted for comparison.

In the sections that follow, inertia weight PSO technique is described in Section 2, LDIW-PSO and the PSO variants adopted for comparison are reviewed in Section 3, parameter settings were experimentally conducted in Section 4, presentations and discussions of the experimental results of LDIW-PSO and its competing variants are made in Section 5, while Section 6 concludes the paper.

## 2. Particle Swarm Optimization

This technique is a simple but efficient population-based, adaptive, and stochastic technique for solving simple and complex optimization problems [17, 18]. It does not need the gradient of the problems to work with, so the technique can be employed for a host of optimization problems. In PSO, a swarm of particles (set of solutions) is randomly positioned (distributed) in the search space. For every particle, the objective function determines the food at its place (value of the objective function). Every particle knows its own actual value of the objective function, its own best value (locally best solution), the best value of the whole swarm (globally best solution), and its own velocity.

PSO maintains a single static population whose members are tweaked (adjust slightly) in response to new discoveries about the space. The method is essentially a form of directed mutation. It operates almost exclusively in multidimensional metric, and usually real-valued, spaces. Because of its origin, PSO practitioners tend to refer to candidate solutions not as a population of individuals but as a swarm of particles. Generally, these particles never die [19], but are moved about in the search space by the directed mutation.

Implementing PSO involves a small number of different parameters that regulates the behavior and efficacy of the algorithm in optimizing a given problem. These parameters are particle swarm size, problem dimensionality, particle velocity, inertia weight, particle velocity limits, cognitive learning rate, social learning rate, and the random factors. The versatility of the usage of PSO comes at a price because for it to work well on any problem at hand, these parameters need tuning and this could be very laborious. The inertia weight parameter (popularly represented as *ω*) has attracted a lot of attentions and seems to be the most important compared with other parameters. The motivation behind its introduction was the desire to better control (or balance) the scope of the (local and global) search and reduce the importance of (or eliminate) velocity clamping, *V*_max⁡_, during the optimization process [20–22]. According to [22], the inertia weight was successful in addressing the former objective, but could not completely eliminate the need for velocity clamping. The feature of the divergence or convergence of particles can be controlled only by parameter *ω*, however, in conjunction with the selection of values for the acceleration constants [22, 23] as well as other parameters.

Each individual in the particle swarm is composed of three *n*-dimension vectors (current position, previous position, and velocity), where *n* is the dimensionality of the search space. Thus, in a physical *n*-dimensional search space, the position and velocity of each particle *i* are represented as the vectors *X*_*i*_ = (*x*_*i*1_,…, *x*_*in*_) and *V*_*i*_ = (*v*_*i*1_,…, *v*_*in*_), respectively. In course of movement in the search space looking for the optimum solution of the problem being optimized, the particle's *velocity* and *position* are updated as follows:

(1) Vik+1=ωVik+c1r1(Pbestik−Xik) +c2r2(Gbestik−Xik),

(2) $$\begin{array}{c} X_{i}^{k+1}=X_{i}^{k}+V_{i}^{k+1}, \end{array}$$

where, *c*_1_ and *c*_2_ are acceleration (weighting) factors known as cognitive and social scaling parameters that determine the magnitude of the random forces in the direction of *P*best (previous best) and *G*best (global previous best); *r*_1_ and *r*_2_ are random numbers between 0 and 1; *k* is iteration index; *ω* is inertia weight. It is common that the positions and velocities of particles in the swarm, when they are being updated, are controlled to be within some specified bounds as shown in Algorithms 1 and 2, respectively. An inertia weight PSO algorithm is shown in Algorithm 3. 

## 3. A Review of LDIW-PSO and Some of Its Competing PSO Variants

Despite the fact that LDIW-PSO algorithm, from the literature, is known to have a shortcoming of premature convergence in solving complex (multipeak) problems, it may not always be true that LDIW-PSO is as weak or inferior as it has been pictured to be by some PSO variants in the literature [2, 7, 13]. Reviewed below are some of these variants and other variants, though not directly compared with LDIW-PSO in the literature, but have been adopted for comparison with LDIW-PSO.

### 3.1. Linear Decreasing Inertia Weight PSO (LDIW-PSO)

The inertia weight parameter was introduced into the original version of PSO by [20]. By introducing a linearly decreasing inertia weight into the original version of PSO, the performance of PSO has been greatly improved through experimental study [24]. In order to further illustrate the effect of this linearly decreasing inertia weight, [4] empirically studied the performance of PSO. With the conviction that a large inertia weight facilitates a global search while a small inertia weight facilitates a local search, a linearly decreasing inertia weight was used with an initial value of 0.9 and a final value of 0.4. By reason of these values, the inertia weight can be interpreted as the fluidity of the medium in which a particle moves [21], showing that setting it to a relatively high initial value (e.g., 0.9) makes particles move in a low viscosity medium and performs extensive exploration. Gradually reducing it to a much lower value (e.g., 0.4) makes the particle moves in a high viscosity medium and performs more exploitation. The experimental results in [4] showed that the PSO converged quickly towards the optimal positions but slowed down its convergence speed when it is near the optima. Thus, by using the linearly decreasing inertia weight, the PSO lacks global search ability at the end of run even when the global search ability is required to jump out of the local minimum in some cases. As a result, employing adapting strategy for adjusting the inertia weight was suggested to improve PSO's performance near the optima. Towards achieving this, there are many improvements on LDIW-PSO in the literature [2, 3, 16, 24–26], which have made PSO to perform with varying degree of successes. Represented in (3) is the LDIW:

(3) $$\begin{array}{c} \omega_{t}=\left(\omega_{\text{start}}-\omega_{\text{end}}\right)\left(\frac{T_{\max}-t}{T_{\max}}\right)+\omega_{\text{end}}, \end{array}$$

where *ω*_start_ and *ω*_end_ are the initial and final values of inertia weight, *t* is the current iteration number, *T*_max⁡_ is the maximum iteration number, and *ω*_*t*_ ∈ [0,1] is the inertia weight value in the *t*th iteration.

### 3.2. Chaotic Descending Inertia Weight PSO (CDIW-PSO)

Chaos is a nonlinear dynamic system which is sensitive to the initial value. It has the characteristic of ergodicity and stochastic property. Using the idea of chaotic mapping, CDIW-PSO was proposed by [2] as shown in (5) based on logistic mapping in (4). The goal was to improve on the LDIW-PSO to avoid getting into local optimum in searching process by utilizing the merits of chaotic optimization

(4) $$\begin{array}{c} z_{k+1}=\mu \times z_{k}\times \left(1-z_{k}\right), \end{array}$$

where *μ* = 4 and *z*_*k*_ is the *k*th chaotic number. The map generates values between 0 and 1, provided that the initial value *z*_0_ ∈ (0,1) and that *z*_0_ ∉ (0.0, 0.25, 0.5, 0.75, 1.0):

(5) $$\begin{array}{c} \omega_{t}=\left(\omega_{\text{start}}-\omega_{\text{end}}\right)\left(\frac{T_{\max}-t}{T_{\max}}\right)+\omega_{\text{end}}\times z_{k+1}, \end{array}$$

where *ω*_start_ and *ω*_end_ are the initial and final values of inertia weight, and rand() is a uniform random number in [0,1]. The experimental results in [2] show that CDIW-PSO outperformed LDIW-PSO in all the test problems used in the experiment in terms of convergence precision, quick convergence velocity, and better global search ability.

### 3.3. Random Inertia Weight and Evolutionary Strategy PSO (REPSO)

This variant proposed in [7] used the idea of simulated annealing and the fitness of particles to design another inertia weight represented by (6). A cooling temperature was introduced to adjust the inertia weight based on certain probability to facilitate jumping off local optimal solutions.

It was experimentally proven that REPSO is significantly superior LDIW-PSO in terms of convergent speed and accuracy:

(6) $$\begin{array}{c} \omega_{t}=\{\begin{array}{cc} \alpha_{1}+\frac{r}{2.0}, & p\geq r,\\ \alpha_{2}+\frac{r}{2.0}, & p<r, \end{array} \end{array}$$

where *α*_1_, *α*_1_ ∈ [0,1] are constants with *α*_1_ > *α*_2_ and *r* ∈ *U*[0,1]. The simulated annealing probability is defined as follows:

(7) $$\begin{array}{c} p=\{\begin{array}{cc} 1, & \underset{1\leq i\leq m}{\min}f_{i}^{t-k}\leq \underset{1\leq i\leq m}{\min}f_{i}^{t},\\ \exp\left(-\frac{\min_{1\leq i\leq m}^{}f_{i}^{t-k}-\min_{1\leq i\leq m}^{}f_{i}^{t}}{T_{t}}\right), & \underset{1\leq i\leq m}{\min}f_{i}^{t-k}>\underset{1\leq i\leq m}{\min}f_{i}^{t}, \end{array} \end{array}$$

where *m* is the number of particles, *f*_*i*_^*t*^ is the fitness value of particle *i* in the *t*th iteration, and the adaptive cooling temperature in the *t*th iteration, *T*_*t*_, is defined as shown in (8):

(8) $$\begin{array}{c} T_{t}=\frac{f_{\text{avg}}^{t}}{f_{\text{best}}^{t}}-1, \end{array}$$

where *f*_best_^*t*^ is the current best fitness value, and *f*_avg_^*t*^ which is defined in (9), is the average fitness value in the *t*th iteration:

(9) $$\begin{array}{c} f_{\text{avg}}^{t}=\frac{1}{m}\overset{m}{\underset{i=1}{\sum }}f_{i}^{t}. \end{array}$$

The combined efforts of the simulated annealing idea and fitness variance of particles improved the global search ability of PSO. In all the experiments performed, REPSO was recorded superior to LDIW-PSO in convergence velocity and precision.

### 3.4. Dynamic Adaptive Particle Swarm Optimization (DAPSO)

DAPSO was introduced by [3] with the aim of proffering solution to the PSO premature convergence problem associated with typical multipeak, high dimensional function optimization problems so as to improve its global optimum and convergence speed. A dynamic adaptive strategy was introduced into the variant to adjust the inertia weight value based on the current swarm diversity and congregate degree as well as the impact on the search performance of the swarm. The experimental results recorded showed that DAPSO was more effective compared with LDIW-PSO. The inertia weight formula that was used is represented in (10):

(10) $$\begin{array}{c} \omega_{t}=\omega_{\min}+\left(\omega_{\max}-\omega_{\min}\right)\times F_{t}\times \varphi_{t}, \end{array}$$

where *ω*_min⁡_ and *ω*_max⁡_ are the minimum and maximum inertia weight values, *t* is the current number of iterations, the diversity function *F*_*t*_ and adjustment function *φ*_*t*_, both in the *t*th iteration, are represented in (11) and (12), respectively:

(11) $$\begin{array}{c} F_{t}=1-\frac{2}{\pi }\text{arc}\tan\left(E\right), \end{array}$$

where *E* is the group fitness as shown in (13):

(12) $$\begin{array}{c} \varphi_{t}=e^{\left(-t^{2}/\left(2\sigma^{2}\right)\right)}, \end{array}$$

where *σ* = *T*/3 and *T* are the total numbers of iterations:

(13) $$\begin{array}{c} E=\frac{1}{N}\overset{N}{\underset{i=1}{\sum }}{\left(f\left(x_{i}\right)-f_{\text{avg}}\right)}^{2}, \end{array}$$

where *N* is the swarm size, *f*(*x*_*i*_) is the fitness of particle *i*, and *f*_avg_ represented in (14) is the current average fitness of the swarm:

(14) $$\begin{array}{c} f_{\text{avg}}=\frac{1}{N}\overset{N}{\underset{i=1}{\sum }}f\left(x_{i}\right). \end{array}$$

### 3.5. Adaptive Particle Swarm Optimization (APSO)

This PSO variant was proposed in [5], in which an adaptive mutation mechanism and a dynamic inertia weight were incorporated into the conventional PSO method. These mechanisms were employed to enhance global search ability and convergence speed and to increase accuracy, while the mutation mechanism affected the particle position updating formula, the dynamic inertia weight affected the inertia weight formula shown in (15). Though APSO was not compared with LDIW-PSO, it outperformed all its competitors as evidenced by all the experimental results:

(15) $$\begin{array}{c} \omega_{t}=0.5\left\{1+\tanh\left[\frac{1}{\alpha }\times F\left(P_{gd}^{t}\right)\right]\right\}, \end{array}$$

where *F*(*P*_*gd*_^*t*^) is the fitness of current best solution in the *t*th iteration, and the parameter *α* is predefined which could be set equal to the fitness of the best particle in the initial population. For the updating of the particle's position, when a particle is chosen for mutation, a Gaussian random disturbance was added as depicted in (16):

(16) $$\begin{array}{c} x_{ij}=x_{ij}+M\times \beta_{ij}, \end{array}$$

where *x*_*ij*_ is the *i*th component of the *j*th particle, *β* ^*ij*^ is a random variable with Gaussian distribution with zero mean and unit variance, and *M* is a variable step size which dynamically decreases according to current best solution fitness. *M* is defined in *t*th iteration according to

(17) $$\begin{array}{c} M_{t}=x_{\max}\times \tanh\left[\frac{1}{\alpha }\times F\left(P_{gd}^{t}\right)\right]. \end{array}$$

### 3.6. Dynamic Nonlinear and Dynamic Logistic Chaotic Map PSO (DLPSO2)

In [11], two types of variants were proposed to solve the premature convergence problem of PSO. In this variant, two dynamic nonlinear methods and logistic chaotic map were used to adjust the inertia weight in parallel according to different fitness values. One of the dynamic nonlinear inertia weights is represented by (18) and (19), while the other is represented by (20) and (21). They are meant to achieve tradeoff between exploration and exploitation:

(18) $$\begin{array}{c} dn={dn}_{\min}+\left({dn}_{\max}-{dn}_{\min}\right)\left(\frac{\text{iter}}{{\text{iter}}_{\max}}\right), \end{array}$$

(19) $$\begin{array}{c} \omega =\omega_{\min}+\left(\omega_{\max}-\omega_{\min}\right){\left(\frac{\text{iter}}{{\text{iter}}_{\max}}\right)}^{dn}, \end{array}$$

(20) $$\begin{array}{c} dn={dn}_{\max}-\left({dn}_{\max}-{dn}_{\min}\right)\left(\frac{\text{iter}}{{\text{iter}}_{\max}}\right), \end{array}$$

(21) $$\begin{array}{c} \omega =\omega_{\max}-\left(\omega_{\max}-\omega_{\min}\right){\left(\frac{\text{iter}}{{\text{iter}}_{\max}}\right)}^{dn}, \end{array}$$

where *dn* is the dynamic nonlinear factor, *ω* is the inertia weight, *ω*_max⁡_ and *ω*_min⁡_ are the maximum and minimum values of *ω*, respectively, *dn*_max⁡_ and *dn*_min⁡_ are the maximum and minimum values of *dn*, respectively, and iter and iter_max⁡_ are the current iteration numbers and the maximum iteration number, respectively.

A dynamic logistic chaotic map in (4) was incorporated into the PSO variant inertia weight as shown in (23) to enrich searching behaviors and avoid being trapped into local optima:

(22) $$\begin{array}{c} \alpha =\alpha_{\max}-\left(\alpha_{\max}-\alpha_{\min}\right)\left(\frac{\text{iter}}{{\text{iter}}_{\max}}\right), \end{array}$$

(23) $$\begin{array}{c} \omega =\alpha +\left(1-\alpha \right)\text{Lmap}, \end{array}$$

where *α* is the dynamic chaotic inertia weight adjustment factor, *α*_max⁡_ and *α*_min⁡_ are the maximum and minimum values of *α*, respectively, and Lmap is the result of logistic chaotic map. In this variant, using (19) and (23) was labeled DLPSO1, while using (21) and (23) was captioned DLPSO2.

For the purpose of achieving a balance between global exploration and local exploitation and also avoiding premature convergence, (19), (21), and (23) were mixed together to dynamically adjust the inertia weight of the variant as shown in Algorithm 4, where *f*_*i*_ is the fitness value of particle *i* and *f*_avg_ is the average fitness value of the swarm. Experiments and comparisons showed that the DLPSO2 outperformed several other well-known improved particle swarm optimization algorithms on many famous benchmark problems in all cases.

### 3.7. Discussions

LDIW-PSO is relatively simple to implement and fast in convergence. When [4] experimentally ascertained that LDIW-PSO is prone to premature convergence, especially when solving complex multimodal optimization problems, a new area of research was opened up for improvements on inertia weight strategies in PSO, and LDIW-PSO became a popular yard stick for many other variants.

From the variants described previously, there are ample expectations that they should outperform LDIW-PSO judging by the various additional strategies introduced into the inertia weight strategies used by them. For example, CDIW-PSO introduced chaotic optimization characteristic, REPSO introduced a combined effort of simulated annealing idea and fitness variance of particles, DAPSO introduced a dynamic adaptive strategy based on swarm diversity function, APSO introduced an adaptive mutation to the particle positions and made the inertia weight dynamic based on the best global fitness, while DLPSO2 used different formulas coupled with chaotic mapping. The general aims of remedying the problem of premature convergence by these variants were not achieved, rather they only struggled to move a bit further than LDIW-PSO in trying to optimize the test problems because a total solution to this problem is for an algorithm to escape all possible local optima and obtain the global optimum. With this, it is possible that LDIW-PSO was subjected to settings, for example, the particles velocity limits [24], which were not appropriate for it to operate effectively.

## 4. Testing with Benchmark Problems

To validate the claim in this paper, 6 different experiments were performed for the purpose of comparing the LDIW-PSO with 6 other different PSO variants, namely, AIW-PSO, CDIW-PSO, REPSO, SA-PSO, DAPSO, and APSO. Different experiments, relative to the competing PSO variants, used different set of test problems which were also used to test LDIW-PSO. Brief descriptions of these test problems are given next. Additional information on these problems can be found in [27–29]. The application software was developed in Microsoft Visual C# programming language.

### 4.1. Benchmark Problems

Six well studied benchmark problems in the literature were used to test the performance of LDIW-PSO, AIW-PSO, CDIW-PSO, REPSO, SA-PSO, DAPSO, and APSO. These problems were selected because their combinations were used to validate the competing PSO variants in the respective literature referenced. The test problems are Ackley, Griewank, Rastrigin, Rosenbrock, Schaffer's f6, and Sphere.

The Ackley problem is multimodal and nonseparable. It is a widely used test problem, and it is defined in (24). The global minimum $f_{1}\left(\vec{x}\right)=0$ is obtainable at $\vec{x}=0$, and the number of local minima is not known:

(24) f1(x→)=−20exp⁡(−0.21n∑i=1dxi2) −exp⁡(1n∑i=1dcos⁡⁡(2πxi))+20+e.

The Griewank problem is similar to that of Rastrigin. It is continuous, multimodal scalable, and nonseparable with many widespread local minima regularly distributed. The complexity of the problem increases with its dimensionality. Its global minimum $f_{2}\left(\vec{x}\right)=0$ is obtainable at $\vec{x}$ = 0, and the number of local minima for arbitrary *n* is not known, but in the two-dimensional case, there are some 500 local minima. This problem is represented by

(25) $$\begin{array}{c} f_{2}\left(\vec{x}\right)=\frac{1}{4000}\left(\overset{d}{\underset{i=1}{\sum }}x_{i}^{2}\right)-\left(\overset{d}{\underset{i=1}{\prod }}\cos\left(\frac{x_{i}}{\sqrt{i}}\right)\right)+1. \end{array}$$

The Rastrigin problem represented in (26) is continuous, multimodal, scalable, and separable with many local minima arranged in a lattice-like configuration. It is based on the Sphere problem with the addition of cosine modulation so as to produce frequent local minima. There are about 50 local minima for two-dimensional case, and its global minimum $f_{3}\left(\vec{x}\right)=0$ is obtainable at $\vec{x}=0$:

(26) $$\begin{array}{c} f_{3}\left(\vec{x}\right)=\overset{d}{\underset{i=1}{\sum }}\left(x_{i}^{2}-10\cos\left(2\pi x_{i}\right)+10\right). \end{array}$$

Shown in (27) is the Rosenbrock problem. It is continuous, unimodal, scalable, and nonseparable. It is a classic optimization problem also known as banana function, the second function of De Jong, or extended Rosenbrock function. Its global minimum $f_{4}\left(\vec{x}\right)=0$ obtainable at $\vec{x}=1$, lies inside a long narrow, parabolic shaped valley. Though it looks simple to solve, yet due to a saddle point it is very difficult to converge to the global optimum:

(27) $$\begin{array}{c} f_{4}\left(\vec{x}\right)=\overset{d-1}{\underset{i=1}{\sum }}\left(100{\left(x_{i+1}-x_{i}^{2}\right)}^{2}\right)+{\left(x_{i}-1\right)}^{2}. \end{array}$$

The Schaffer's f6 problem represented in (28) is 2-dimensional, continuous, multimodal, and nonseparable with unknown number of many local minima. Its global minimum $f_{5}\left(\vec{x}\right)=0$ is obtainable at $\vec{x}$ = 0:

(28) $$\begin{array}{c} f_{5}\left(\vec{x}\right)=\overset{d-1}{\underset{i=1}{\sum }}\left(0.5+\frac{\sin^{\text{2}}\left(\sqrt{x_{i+1}^{2}+x_{i}^{2}}\right)-0.5}{{\left(0.001\left(x_{i+1}^{2}+x_{i}^{2}\right)+1\right)}^{2}}\right). \end{array}$$

The Sphere problem also known as the first De Jong function is continuous, convex, unimodal, scalable, and separable. It is one of the simplest test benchmark problems. Its global minimum $f_{6}\left(\vec{x}\right)=0$ is obtainable at $\vec{x}$ = 0, and the problem is represented by

(29) $$\begin{array}{c} f_{6}\left(\vec{x}\right)=\overset{d}{\underset{i=1}{\sum }}x_{i}^{2}. \end{array}$$

### 4.2. Parameter Settings

The limits of particle velocity could negatively affect the performance of PSO algorithm if it is not properly set. As a result, different work has been done to determine the velocity limits of particles in order to improve on the performance of PSO. Researches in this direction are [4, 24, 30] the three major methods that appear in the literature, for computing the velocity clamping (*V*_min⁡_ and *V*_max⁡_) are (i) multiplying the search space range with certain percentage (*δ*); that is, *V*_max⁡_ = *δ*(*X*_max⁡_ − *X*_min⁡_) and *V*_min⁡_ = −*V*_max⁡_; (ii) multiplying both the minimum and maximum limits of the search space separately with certain percentage (*δ*); that is, *V*_max⁡_ = *δ*(*X*_max⁡_) and *V*_min⁡_ = *δ*(*X*_min⁡_); (iii) assigning the search space upper limit to *V*_max⁡_. It is obvious from (i) and (ii) that the parameter *δ* is very important. As a result, different values have been used by different authors [5, 6, 13, 30] for *δ* to determine velocity clamping for particles.

In trying to substantiate the fact that LDIW-PSO is not as weak or inferior as many authors claimed it to be, an experiment was conducted to investigate the effect of the parameter *δ* on the performance of LDIW-PSO using the benchmark problems described previously. The results were used as a guide to set *δ* in LDIW-PSO before embarking on some experimental comparison, between it and some other PSO variants described previously to prove that LDIW-PSO is superior to many of the variants that have been claimed to be better that it. The results of the experiments are listed in the Appendix. Using the results as guide, the value of *δ* was set in LDIW-PSO for the various test problems as listed in Table 1. However, *δ* was set to 0.015 for *f*_2_ in Experiment 2 and 0.25 for *f*_3_ in Experiments 2 and 5.

### 4.3. Experimental Setup

The settings for the different experiments carried out for the comparisons are described next one after the other. In all the experiments, LDIW-PSO was subjected to the settings of its competitors as recorded in the literature. For LDIW-PSO, *c*_1_ = *c*_2_ = 2.0, *ω*_max⁡_ = 0.9, *ω*_min⁡_ = 0.4, *V*_min⁡_ = *δX*_min⁡_, and *V*_max⁡_ = *δX*_max⁡_.  Table 2 shows the respective search and initialization ranges for all the algorithms, the symbol “–” means that the corresponding test problem was not used by the algorithm under which the symbol appears.

*Experiment  1.* The purpose of this experiment was to compare LDIW-PSO with CDIW-PSO [2]. All the test problems described previously were used in this experiment, except *f*_1_. The dimension for *f*_5_ was 2, while it was 30 for others. The maximum numbers of iterations were set to 1500 with swarm size of 20, and the experiment was repeated 500 times. Stated in Table 3 are the set goals (criteria) of success for each of the problems.

*Experiment  2*. The purpose of this experiment was to compare LDIW-PSO with REPSO [7]. All the test problems in Table 1 except *f*_1_ were used. The dimension for *f*_5_ was 2, while it was 10 for others. The performances of the algorithms were considered at different number of iterations as shown in Section 5, in line with what is recorded in the literature [7]. The swarm size used was 30, and the experiment was repeated 50 times. 

*Experiment  3.* The purpose of this experiment was to compare LDIW-PSO with DAPSO [13]. Test problems *f*_1_ − *f*_3_ were used with four different problem dimensions of 20, 30, 40, and 50. The maximum number of iterations and swarm size was set to 3000 and 30, respectively, and the experiment was repeated 50 times.

*Experiment  4*. The purpose of this experiment was to compare LDIW-PSO with APSO [5]. *f*_2_, *f*_3_, and *f*_4_ were used as test problems with three different problem dimensions of 10, 20, and 30. The respective maximum numbers of iterations associated with these dimensions are 1000, 1500, and 2000, respectively. The experiment was carried out over three different swarm sizes, 20, 40, and 80 for each problem dimension, and the experiment was repeated 30 times.

*Experiment  5.* This experiment compared LDIW-PSO with DLPSO2 [11]. All the test problems except *f*_4_ were used in the experiment with two different problem dimensions of 2 (for *f*_3_ and *f*_5_) and 30 (for *f*_1_, *f*_2_, and *f*_6_). The maximum number of iterations was set to 2000 and swarm sizes to 20, and the experiment was repeated 20 times. 

## 5. Results and Discussions

Presented in Tables 4–8 are the results obtained for all the experiments. The results for all the competing PSO variants were obtained from the respective referenced papers, and they are presented here the way they were recorded. Thus, the recording of the results for LDIW-PSO was patterned after them. In each of the tables, bold values represent the best results. In the tables, mean best fitness (solution) is a measure of the precision that the algorithm can get within a given number of iterations, standard deviation (Std. Dev.) is a measure of the algorithm's stability and robustness, while success rate (SR) [31] is the rate at which an algorithm obtains optimum fitness result in the criterion range during a given number of independent runs.

*Experiment  1 (comparison of LDIW-PSO with CDIW-PSO). *The results in Table 4 clearly reveal a great difference in performance between LDIW-PSO and CDIW-PSO [2]. The results are compared based on the final accuracy of the averaged best solutions, success rate (SR), and standard deviation (Std. Dev.) of the best solutions. In all the test problems, the result indicates that LDIW-PSO can get better optimum fitness result, showing better convergence precision. LDIW-PSO is also more stable and robust compared with CDIW-PSO, because its standard deviation is comparatively lesser in three of the test problems. Besides, LDIW-PSO has better global search ability and could easily get out of local optima than CDIW-PSO.

*Experiment  2 (comparison of LDIW-PSO with REPSO). *In Table 5, the comparison between LDIW-PSO and REPSO was based on the final accuracy of the averaged best solutions relative to the specified number of iterations and convergence speed as recorded in [7]. From the results, REPSO appears to converge faster in Griewank and Rastrigin at the beginning but was overtaken by LDIW-PSO which eventually converged faster and had better accuracy. In Rosenbrock and Sphere problems, LDIW-PSO had better convergence speed and accuracy in comparison with REPSO. The symbol “—” means that the corresponding iteration number was not considered for the test problem under which the symbol appears.

*Experiment  3 (comparison of LDIW-PSO with DAPSO)*. The results for DAPSO were obtained from [13]. Comparing these results with that of LDIW-PSO were measured using the final accuracy of the respective mean best solutions across the different problems dimensions as shown in Table 6. In all the problems and dimensions except dimension 40 of Rastrigin, LDIW-PSO outperformed DAPSO getting better fitness quality and precision. This is a clear indication that LDIW-PSO has better global search ability and is not easily trapped in local optima compared with DAPSO.

*Experiment  4 (comparison of LDIW-PSO with APSO)*. Recorded in Table 7 are the results for LDIW-PSO and APSO [5] over different swarm sizes, dimensions, and iterations. The basis for comparison is the final accuracy and quality of their mean best fitness. The two variants put up a good competition. In Griewank and Rastrigin, APSO performed better in smaller dimensions, while LDIW-PSO performed better in higher dimensions. But in Rosenbrock, LDIW-PSO outperformed APSO in locating better solutions to the problem.

*Experiment  5 (comparison of LDIW-PSO with DLPSO2)*. The results for LIDIW-PSO and DLPSO2 [11] in Table 8 are compared based on the best fitness, mean best fitness, convergence speed, as well as standard deviation (Std. Dev.) of the best solutions. In Rastrigin, the two algorithms have equal performances. However, in other test problems, the result indicates that LDIW-PSO can get better optimum fitness result, showing better convergence speed. LDIW-PSO is also more stable and robust compared with DLPSO2, because its standard deviation is comparatively smaller in all the test problems. Besides, LDIW-PSO demonstrated better global search ability and getting out of local optima than DLPSO2.

## 6. Conclusion

Motivated by the superiority claims by some PSO variants over LDIW-PSO in terms of performance, a number of experiments were performed in this paper to empirically verify some of these claims. Firstly, an appropriate (approximate) percentage of the test problems search space limits were obtained to determine the particle velocity limits for LDIW-PSO. Secondly, these values were used in the implementation of LDIW-PSO for some benchmark optimization problems and the results obtained compared with that of some PSO variants that have previously claimed superiority in performance. LDIW-PSO performed better than these variant. The performances of the two other recent PSO variants with different inertia weight strategies were also compared with LDIW-PSO on similar problems with the latter showing competitive advantage. This work has therefore showed that with good experimental setting, LDIW-PSO will perform competitively with similar variants. Precious claims of inferior performance might therefore be due to some unfavourable experimental settings. The Appendix provides further simulation results that can provide useful hints for deciding the setting velocity threshold for particles for LDIW-PSO.

## Figures and Tables

**Algorithm 1:**
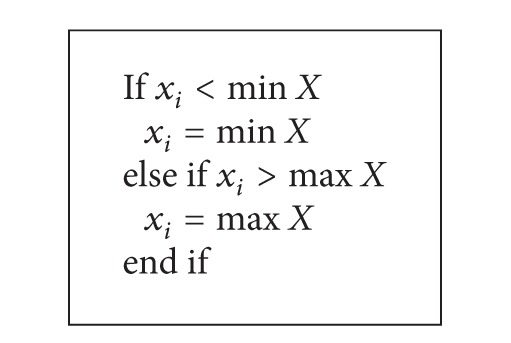
Particle position clamping.

**Algorithm 2:**
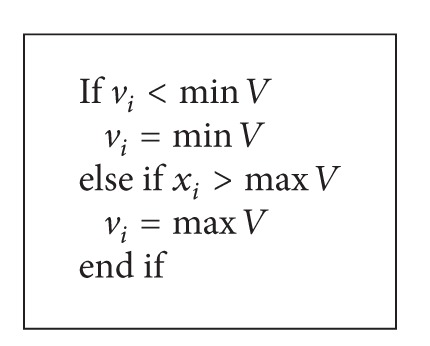
Particle velocity clamping.

**Algorithm 3:**
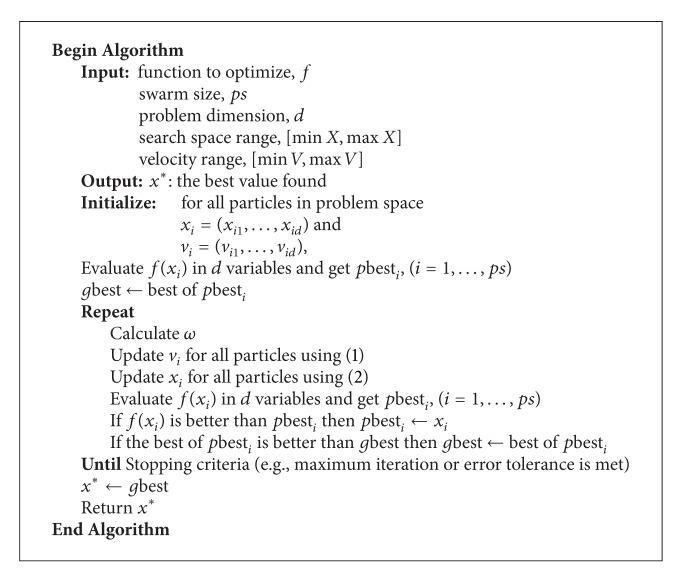
Inertia weight PSO algorithm.

**Algorithm 4:**
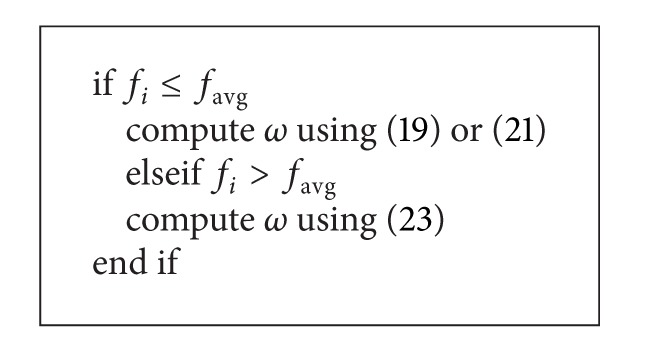


**Table 1:** Settings for parameter *δ* in LDIW-PSO.

Problem	*f*_1_	*f*_2_	*f*_3_	*f*_4_	*f*_5_	*f*_6_
*δ*	0.05	0.0075	0.05	0.015	0.075	0.015

**Table 2:** Test problems search and initialization ranges for the PSO variants.

Label	CDIW-PSO	REPSO	DAPSO	APSO	DLPSO2
*f*_1_	—	—	[−32,32]	—	[−32,32]
*f*_2_	[−600,600]	[−600,600]	[−600,600]	[−600,600]	[−600,600]
*f*_3_	[−5.12,5.12]	[−10,10]	[−5.12,5.12]	[−5.12,5.12]	[−10,10]
*f*_4_	[−30,30]	[−100,100]	—	[−30,30]	—
*f*_5_	[−100,100]	[−10,10]	—	—	[−1.0,1.0]
*f*_6_	[−100,100]	[−10,10]	—	—	[−100,100]

**Table 3:** Goals for the test problems in CDIW-PSO.

Label	*f*_2_	*f*_3_	*f*_4_	*f*_5_	*f*_6_
Goal	0.05	50.0	100.0	0.00001	0.01

**Table 4:** Experimental results for LDIW-PSO compared with CDIW-PSO.

Criteria	Griewank		Rastrigin		Rosenbrock		Schaffer's f6		Sphere	
	CDIW-PSO	LDIW-PSO	CDIW-PSO	LDIW-PSO	CDIW-PSO	LDIW-PSO	CDIW-PSO	LDIW-PSO	CDIW-PSO	LDIW-PSO
Mean fitness	0.014773	**0.007609**	40.044561	**33.055877**	44.305058	**31.148789**	0.007732	**0.000117**	0.000092	**0.000000**
Std. Dev.	0.002959	**0.008439**	**8.028912**	10.498048	**8.861012**	20.832263	0.001546	**0.001058**	0.000016	**0.000000**
SR (%)	96.2	**100**	83.6	**92.8**	**99.6**	98.0	22.0	**98.6**	100	100

**Table 5:** Experimental results for LDIW-PSO compared with REPSO.

Iteration	Griewank^1^		Rastrigin		Rosenbrock^2^		Sphere	
	REPSO	LDIW-PSO	REPSO	LDIW-PSO	REPSO	LDIW-PSO	REPSO	LDIW-PSO
50	—	—	—	—	—	—	—	—
100	**0.6705**	0.7859	**30.7320**	44.2732	—	—	0.00671	**0.00493**
200	**0.4922**	0.6437	—	—	—	—	—	—
300	**0.2487**	0.5607	—	—	—	—	2.1142**e** − 04	2.9792*e* − 04
400	**0.2345**	0.4318	20.6671	**16.5414**	—	—	—	—
500	**0.1658**	0.3185	17.3751	**10.4621**	570.7681	**352.1663**	7.1144*e* − 05	9.1853**e** − 07
800	—	—	15.5611	**3.9143**	—	—	6.8751*e* − 06	5.8431**e** − 17
1000	0.1461	**0.0967**	10.8120	**3.2609**	300.1407	**218.9924**	5.6367*e* − 07	1.2425**e** − 28
1500	0.1353	**0.0842**	—	—	260.8421	**138.2756**	—	—
2000	0.1089	**0.0794**	—	—	170.2157	**79.9941**	—	—
3000	—	—	—	—	60.4418	**21.5586**	—	—

^1^This problem is slightly different from the one in (25).

^2^This problem is slightly different from the one in (27).

**Table 6:** Experimental results for LDIW-PSO compared with DAPSO.

Dim	Ackley		Griewank		Rastrigin	
	DAPSO	LDIW-PSO	DAPSO	LDIW-PSO	DAPSO	LDIW-PSO
20	3.906209*e* − 014	8.970602**e** − 015	8.605280*e* − 002	1.649481**e** − 002	2.159059*e* + 001	2.040020**e** + 001
30	4.159541*e* − 008	1.527799**e** − 010	2.583338*e* − 002	9.258783**e** − 003	3.263463*e* + 001	2.996404**e** + 001
40	7.046093*e* − 005	2.578715**e** − 007	1.087868*e* − 002	4.875733**e** − 003	3.890287**e** + 001	4.109865*e* + 001
50	1.025568*e* − 003	1.629095**e** − 005	1.346732*e* − 002	4.335978**e** − 003	4.823559*e* + 001	4.606947**e** + 001

**Table 7:** Experimental results for LDIW-PSO compared with APSO.

Swarm size	Dim	Maximum iteration	Griewank		Rastrigin		Rosenbrock	
			APSO	LDIW-PSO	APSO	LDIW-PSO	APSO	LDIW-PSO
20	10	1000	**0.0983**	0.2347	**5.1565**	12.4602	5.8467	**4.3695**
	20	1500	0.0237	**0.0150**	**16.0456**	27.6708	47.9842	**19.1223**
	30	2000	0.0117	**0.0103**	42.2325	**33.2050**	100.4528	**29.3482**

40	10	1000	**0.0952**	0.2231	**2.9468**	10.5713	4.5431	**3.9145**
	20	1500	**0.0201**	0.0211	**15.3678**	19.3199	38.3464	**16.5186**
	30	2000	0.0105	**0.0099**	33.7538	**26.3453**	72.5473	**26.9638**

80	10	1000	**0.0689**	0.1294	**2.0457**	9.0800	**4.1680**	6.5127
	20	1500	0.0199	**0.0184**	**10.0563**	16.4368	27.9547	**17.6043**
	30	2000	0.0102	**0.0080**	25.3473	**23.2303**	69.0609	**24.6653**

**Table 8:** Experimental results for LDIW-PSO compared with DLPSO2.

Criteria	Best fitness	Mean fitness	Std. Dev.
Ackley			
DLPSO2	8.6209*e* − 06	0.4743	0.6527
LDIW-PSO	2.0441**e** − 07	**0.0000**	**0.0000**
Griewank			
DLPSO2	7.7589*e* − 06	0.0086	0.0114
LDIW-PSO	3.5694**e** − 13	**0.0083**	**0.0088**
Rastrigin			
DLPSO2	−2	−2	0
LDIW-PSO	−2	−2	0
Schaffer's f6			
DLPSO2	7.5206*e* − 07	5.6300*e* − 06	2.8969*e* − 06
LDIW-PSO	0.0000**e** + 00	0.0000**e** + 00	0.0000**e** + 00
Sphere			
DLPSO2	7.6941*e* − 06	9.5001*e* − 06	4.9557*e* − 07
LDIW-PSO	4.1289**e** − 14	0.0000**e** + 00	0.0000**e** + 00

**Table 9:** Different values of parameter *δ* and respective mean best fitness for Griewank test problem.

*δ*	Dimension 10		Dimension 30		Dimension 50	
	Size = 20	Size = 30	Size = 20	Size = 30	Size = 20	Size = 30
1.0	9.913*e* − 02	9.125*e* − 02	1.157*e* + 01	5.607*e* + 00	6.269*e* + 01	3.941*e* + 01
0.75	9.645*e* − 02	8.825*e* − 02	3.088*e* + 00	1.451*e* − 02	1.519*e* + 01	6.875*e* + 00
0.5	9.983*e* − 02	9.018*e* − 02	1.972*e* − 01	1.601*e* − 02	2.003*e* + 00	5.522*e* − 01
0.25	1.002*e* − 01	2.925**e** − 02	1.602*e* − 02	1.458*e* − 02	1.200*e* − 02	9.885*e* − 03
0.15	9.772*e* − 02	9.276*e* − 02	1.556*e* − 02	1.450*e* − 02	9.925*e* − 03	8.654*e* − 03
0.1	1.044*e* − 01	9.141*e* − 02	1.489*e* − 02	1.564*e* − 02	1.027*e* − 02	9.339*e* − 03
0.075	1.064*e* − 01	1.006*e* − 01	1.328*e* − 02	1.389*e* − 02	8.937*e* − 03	7.963*e* − 03
0.05	1.011*e* − 01	9.417*e* − 02	1.521*e* − 02	1.580*e* − 02	8.224*e* − 03	7.821*e* − 03
0.025	9.682*e* − 02	8.738*e* − 02	1.604*e* − 02	1.668*e* − 02	7.108*e* − 03	7.354*e* − 03
0.015	9.028**e** − 02	8.648*e* − 02	1.379*e* − 02	1.444*e* − 02	5.719*e* − 03	6.226*e* − 03
0.01	1.274*e* − 01	1.265*e* − 01	1.148*e* − 02	1.141*e* − 02	5.005*e* − 03	4.768*e* − 03
0.0075	2.251*e* − 01	2.078*e* − 01	7.160**e** − 03	7.595**e** − 03	4.237*e* − 03	4.021**e** − 03
0.005	5.546*e* − 01	3.751*e* − 01	8.006*e* − 03	8.030*e* − 03	4.025**e** − 03	4.526*e* − 03
0.0025	1.258*e* + 00	6.833*e* − 01	1.203*e* − 02	1.218*e* − 02	6.808*e* − 03	6.013*e* − 03
0.0015	1.895*e* + 01	9.642*e* − 01	1.415*e* − 02	1.434*e* − 02	7.226*e* − 03	7.419*e* − 03
0.001	4.061*e* + 00	2.083*e* + 00	1.366*e* − 02	1.622*e* − 02	7.184*e* − 03	7.462*e* − 03

**Table 10:** Different values of parameter *δ* and respective mean best fitness for Rastrigin test problem.

*δ*	Dimension 10		Dimension 30		Dimension 50	
	Size = 20	Size = 30	Size = 20	Size = 30	Size = 20	Size = 30
1.0	4.551*e* + 00	3.400**e** + 00	9.959*e* + 01	8.462*e* + 01	2.694*e* + 02	2.361*e* + 02
0.75	4.537**e** + 00	3.619*e* + 00	6.924*e* + 01	5.866*e* + 01	1.935*e* + 02	1.729*e* + 02
0.5	4.646*e* + 00	3.476*e* + 00	5.253*e* + 01	4.282*e* + 01	1.330*e* + 02	1.151*e* + 02
0.25	6.484*e* + 00	5.247*e* + 00	4.534*e* + 01	4.197*e* + 01	8.943*e* + 01	8.462*e* + 01
0.15	1.043*e* + 01	9.013*e* + 00	4.142*e* + 01	3.798*e* + 01	7.204*e* + 01	6.590*e* + 01
0.1	1.149*e* + 01	9.470*e* + 00	3.702*e* + 01	3.380*e* + 01	6.183*e* + 01	5.653*e* + 01
0.075	1.077*e* + 01	9.397*e* + 00	3.328*e* + 01	2.917*e* + 01	5.394*e* + 01	4.824*e* + 01
0.05	1.162*e* + 01	1.022*e* + 01	3.302**e** + 01	2.943**e** + 01	5.370**e** + 01	4.704**e** + 01
0.025	1.373*e* + 01	1.160*e* + 01	3.607*e* + 01	3.194*e* + 01	5.474*e* + 01	4.860*e* + 01
0.015	1.387*e* + 01	1.159*e* + 01	3.893*e* + 01	3.521*e* + 01	5.762*e* + 01	5.087*e* + 01
0.01	1.431*e* + 01	1.221*e* + 01	4.010*e* + 01	3.565*e* + 01	5.995*e* + 01	5.390*e* + 01
0.0075	1.475*e* + 01	1.213*e* + 01	4.164*e* + 01	3.692*e* + 01	6.256*e* + 01	5.476*e* + 01
0.005	1.868*e* + 01	1.398*e* + 01	4.300*e* + 01	3.663*e* + 01	6.451*e* + 01	5.464*e* + 01
0.0025	3.337*e* + 01	2.507*e* + 01	7.294*e* + 01	4.917*e* + 01	9.215*e* + 01	6.073*e* + 01
0.0015	4.794*e* + 01	4.027*e* + 01	1.168*e* + 02	7.803*e* + 01	1.396*e* + 02	8.922*e* + 01
0.001	5.792*e* + 01	5.220*e* + 01	1.898*e* + 02	1.548*e* + 02	2.102*e* + 02	1.390*e* + 02

**Table 11:** Different values of parameter *δ* and respective mean best fitness for Rosenbrock test problem.

*δ*	Dimension 10		Dimension 30		Dimension 50	
	Size = 20	Size = 30	Size = 20	Size = 30	Size = 20	Size = 30
1.0	1.165*e* + 04	1.040*e* + 04	1.851*e* + 05	2.873*e* + 04	3.075*e* + 06	1.148*e* + 06
0.75	6.020*e* + 03	4.020*e* + 03	2.009*e* + 04	1.711*e* + 04	8.240*e* + 05	1.837*e* + 05
0.5	2.585*e* + 03	2.189*e* + 03	1.128*e* + 04	8.214*e* + 03	1.175*e* + 04	1.360*e* + 04
0.25	1.872*e* + 01	5.571*e* + 00	4.307*e* + 02	4.445*e* + 02	2.315*e* + 03	1.056*e* + 03
0.15	1.075*e* + 01	4.229*e* + 00	4.910*e* + 01	4.750*e* + 01	1.156*e* + 02	9.710*e* + 01
0.1	4.798*e* + 00	4.241*e* + 00	4.248*e* + 01	4.147*e* + 01	9.217*e* + 01	8.699*e* + 01
0.075	4.680**e** + 00	4.099**e** + 00	4.531*e* + 01	3.607*e* + 01	1.073*e* + 02	7.701*e* + 01
0.05	5.182*e* + 00	4.534*e* + 00	3.453*e* + 01	3.282*e* + 01	6.858*e* + 01	6.383*e* + 01
0.025	5.770*e* + 00	5.598*e* + 00	3.148*e* + 01	3.035*e* + 01	5.450*e* + 01	5.215*e* + 01
0.015	7.818*e* + 00	6.800*e* + 00	2.956**e** + 01	2.832**e** + 01	5.207**e** + 01	5.218*e* + 01
0.01	7.748*e* + 00	6.480*e* + 00	2.962*e* + 01	2.891*e* + 01	5.487*e* + 01	5.154**e** + 01
0.0075	8.085*e* + 00	7.945*e* + 00	2.998*e* + 01	2.948*e* + 01	5.505*e* + 01	5.164*e* + 01
0.005	6.491*e* + 00	6.896*e* + 00	3.134*e* + 01	3.015*e* + 01	5.544*e* + 01	5.263*e* + 01
0.0025	7.943*e* + 01	7.682*e* + 00	3.052*e* + 01	2.915*e* + 01	5.656*e* + 01	5.163*e* + 01
0.0015	5.003*e* + 01	1.408*e* + 01	3.095*e* + 01	2.672*e* + 01	5.398*e* + 01	5.174*e* + 01
0.001	2.417*e* + 04	3.426*e* + 03	3.020*e* + 01	2.949*e* + 01	5.614*e* + 01	5.222*e* + 01

**Table 12:** Different values of parameter *δ* and respective mean best fitness for Sphere test problem.

*δ*	Dimension 10		Dimension 30		Dimension 50	
	Size = 20	Size = 30	Size = 20	Size = 30	Size = 20	Size = 30
1.0	1.043*e* − 20	3.679*e* − 23	1.140*e* + 03	5.400*e* + 02	7.380*e* + 03	4.400*e* + 03
0.75	9.490*e* − 21	1.554*e* − 23	1.600*e* + 02	4.000*e* + 01	1.460*e* + 03	7.600*e* + 02
0.5	5.108*e* − 21	1.048*e* − 23	1.349*e* − 08	4.015*e* − 10	1.000*e* + 02	2.000*e* + 01
0.25	8.561*e* − 22	5.859*e* − 24	3.547*e* − 09	6.110*e* − 11	1.538*e* − 05	4.976*e* − 07
0.15	5.304*e* − 21	9.144*e* − 25	1.503*e* − 09	2.963*e* − 11	6.952*e* − 06	2.114*e* − 07
0.1	6.679*e* − 23	1.203*e* − 24	4.432*e* − 10	1.193*e* − 11	2.224*e* − 06	7.656*e* − 08
0.075	8.577*e* − 23	2.149*e* − 25	2.397*e* − 10	8.813*e* − 12	1.306*e* − 06	4.954*e* − 08
0.05	3.921*e* − 23	1.794*e* − 25	1.147*e* − 10	3.490*e* − 12	5.098*e* − 07	2.235*e* − 08
0.025	1.006*e* − 23	4.835*e* − 26	2.596*e* − 11	7.592*e* − 13	1.620*e* − 07	6.654*e* − 09
0.015	2.466*e* − 24	1.795*e* − 26	1.349*e* − 11	2.364*e* − 13	5.689*e* − 08	2.222*e* − 09
0.01	1.022*e* − 24	4.326*e* − 27	3.998*e* − 12	1.245*e* − 13	3.983*e* − 08	8.837*e* − 10
0.0075	9.942*e* − 25	3.991*e* − 27	2.758*e* − 12	7.017*e* − 14	1.115*e* − 08	5.786*e* − 10
0.005	6.363**e** − 25	2.300**e** − 27	1.449*e* − 12	3.061*e* − 14	1.116*e* − 08	2.034*e* − 10
0.0025	2.003*e* − 23	1.376*e* − 26	3.638**e** − 13	9.420**e** − 15	1.592*e* − 09	6.778*e* − 11
0.0015	4.469*e* − 08	2.962*e* − 08	7.378*e* − 13	1.254*e* − 14	1.062**e** − 09	3.130*e* − 11
0.001	2.900*e* + 02	9.887*e* + 01	5.711*e* − 02	8.265*e* − 13	2.563*e* − 09	2.755**e** − 11

**Table 13:** Different values of parameter *δ* and respective mean best fitness for Schaffer's f6 test problem.

*δ*	Dimension 2	
	Size = 20	Size = 30
1.0	1.342*e* − 03	5.446*e* − 04
0.75	2.392*e* − 03	9.335*e* − 04
0.5	2.052*e* − 03	7.651*e* − 04
0.25	1.387*e* − 03	7.212*e* − 04
0.15	7.756*e* − 04	2.731*e* − 04
0.1	6.816*e* − 04	1.847*e* − 04
0.075	4.865**e** − 04	1.749*e* − 04
0.05	6.413*e* − 04	1.612**e** − 04
0.025	4.275*e* − 03	2.740*e* − 03
0.015	5.625*e* − 03	3.129*e* − 03
0.01	4.726*e* − 03	2.993*e* − 03
0.0075	4.594*e* − 03	2.683*e* − 03
0.005	5.663*e* − 03	3.327*e* − 03
0.0025	5.940*e* − 03	4.760*e* − 03
0.0015	7.582*e* − 03	5.449*e* − 03
0.001	7.776*e* − 03	6.092*e* − 03

## Appendix

Tables 9, 10, 11, 12, and 13 show the results of LDIW-PSO in optimizing some benchmark problems so as to determine a suitable value for *δ* that was used to set the velocity limits for the particles. The experiments were repeated 500 times for each of the problems. Two different swarm sizes of 20 and 30 were used for each of the three different problem dimensions 10, 30, and 50. The respective number of iterations that was used with the dimensions is 1000, 1500, and 2000. The LDIW strategy was decreased from 0.9 to 0.4 in course of searching for solution to the problem [7, 10–12, 27], the acceleration constants (*c*_1_ and *c*_2_) were set to 2.0, and *V*_max⁡_ = *δ*(*X*_max⁡_) and *V*_min⁡_ = *δ*(*X*_min⁡_). In the tables, bold values represent the best mean fitness value.

## References

[1] Eberhart RC, Kennedy J New optimizer using particle swarm theory.

[2] Feng Y, Teng GF, Wang AX, Yao YM Chaotic inertia weight in particle swarm optimization.

[3] Xin J, Chen G, Hai Y A particle swarm optimizer with multi-stage linearly-decreasing inertia weight.

[4] Shi YH, Eberhart RC Empirical study of particle swarm optimization.

[5] Alfi A (2011). PSO with adaptive mutation and inertia weight and its application in parameter estimation of dynamic systems. *Acta Automatica Sinica*.

[6] Chen G, Huang X, Jia J, Min Z Natural exponential inertia weight strategy in particle swarm optimization.

[7] Gao Y-L, Duan Y-H A new particle swarm optimization algorithm with random inertia weight and evolution strategy.

[8] Gao Y, An X, Liu J A particle swarm optimization algorithm with logarithm decreasing inertia weight and chaos mutation.

[9] Kentzoglanakis K, Poole M Particle swarm optimization with an oscillating inertia weight.

[10] Li HR, Gao YL Particle swarm optimization algorithm with exponent decreasing inertia weight and stochastic mutation.

[11] Liu H, Su R, Gao Y, Xu R Improved particle swarm optimization using two novel parallel inertia weights.

[12] Malik RF, Rahman TA, Hashim SZM, Ngah R (2007). New particle swarm optimizer with sigmoid increasing inertia weight. *International Journal of Computer Science and Security*.

[13] Shen X, Chi Z, Yang J, Chen C, Chi Z Particle swarm optimization with dynamic adaptive inertia weight.

[14] Jian-Ru Z, Guo-Li Z, Hua Z Hybrid linear and nonlinear weight particle swarm optimization algorithm.

[15] Chauhan P, Deep K, Pant M (2013). Novel inertia weight strategies for particle swarm optimization. *Memetic Computing*.

[16] Shi Y, Eberhart RC Fuzzy adaptive particle swarm optimization.

[17] Arasomwan MA, Adewumi AO An adaptive velocity particle swarm optimization for high-dimensional function optimization.

[18] Arasomwan MA, Adewumi AO On adaptive chaotic inertia weights in particle swarm optimization.

[19] Luke S (2011). *Essentials of Metaheuristics, a Set of Undergraduate Lecture Notes*.

[20] Shi YH, Eberhart RC A modified particle swarm optimizer.

[21] Poli R, Kennedy J, Blackwell T (2007). Particle swarm optimization: an overview. *Swarm Intelligence*.

[22] Engelbrecht AP (2007). *Computational Intelligence: An Introduction*.

[23] Iwasaki N, Yasuda K, Ueno G (2006). Dynamic parameter tuning of particle swarm optimization. *IEEJ Transactions on Electrical and Electronic Engineering*.

[24] Shi Y, Eberhart R, Porto VW, Saravanan N, Waagen D, Eiben AE (1998). Parameter selection in particle swarm optimization. *Evolutionary Programming VII*.

[25] Eberhart RC, Shi Y Tracking and optimizing dynamic systems with particle swarms.

[26] Nickabadi A, Ebadzadeh MM, Safabakhsh R (2011). A novel particle swarm optimization algorithm with adaptive inertia weight. *Applied Soft Computing Journal*.

[27] Molga M, Smutnicki C Test functions for optimization needs. http://www.zsd.ict.pwr.wroc.pl/files/docs/functions.pdf.

[28] Montaz AM, Khompatraporn C, Zabinsky ZB (2005). A numerical evaluation of several stochastic algorithms on selected continuous global optimization test problems. *Journal of Global Optimization*.

[29] Chetty S, Adewumi AO (2013). Three new stochastic local search algorithms for continuous optimization problems. *Computational Optimization and Applications*.

[30] Evers GI (2009). *An automatic regrouping mechanism to deal with stagnation in particle swarm optimization [M.S. thesis]*.

[31] Sawyerr BA, Ali MM, Adewumi AO (2011). A comparative study of some real-coded genetic algorithms for unconstrained global optimization. *Optimization Methods and Software*.

